# Management of intrahepatic cholangiocarcinoma in a patient with Caroli's disease: a case report and literature review

**DOI:** 10.1093/jscr/rjaf380

**Published:** 2025-08-26

**Authors:** Omar Abouelwafa, Nadja Lehwald-Tywuschik, Dimitrios Prassas, Wolfram Trudo Knoefel, Sascha Vaghiri

**Affiliations:** Department of Surgery (A), Heinrich-Heine-University, Medical Faculty and University Hospital Duesseldorf, Moorenstr. 5, 40225 Duesseldorf, North Rhine-Westphalia, Germany; Department of Surgery, AKH Viersen, Hoserkirchweg 63, 41747 Viersen, North Rhine-Westphalia, Germany; Department of Surgery (A), Heinrich-Heine-University, Medical Faculty and University Hospital Duesseldorf, Moorenstr. 5, 40225 Duesseldorf, North Rhine-Westphalia, Germany; Department of Surgery, Katholisches Klinikum Essen, Philippusstift, Teaching Hospital of Duisburg-Essen University, Huelsmannstr, 17, 45355 Essen, North Rhine-Westphalia, Germany; Department of Surgery (A), Heinrich-Heine-University, Medical Faculty and University Hospital Duesseldorf, Moorenstr. 5, 40225 Duesseldorf, North Rhine-Westphalia, Germany; Department of Surgery (A), Heinrich-Heine-University, Medical Faculty and University Hospital Duesseldorf, Moorenstr. 5, 40225 Duesseldorf, North Rhine-Westphalia, Germany

**Keywords:** Caroli's disease, intrahepatic cholangiocarcinoma, cystic dilatation, biliary cirrhosis, left hepatectomy, liver transplantation

## Abstract

Caroli's disease is a rare congenital disorder characterized by cystic dilatations of the intrahepatic bile ducts, often leading to recurrent bacterial cholangitis, biliary lithiasis, and cirrhosis. In 7%–16% of cases, it progresses to intrahepatic cholangiocarcinoma, emphasizing the need for early diagnosis and intervention. This report presents a 56-year-old patient with recurrent upper abdominal pain, chronic fatigue, and night sweats. Imaging studies (computed tomography, endoscopic retrograde cholangiopancreatography, magnetic resonance cholangiopancreatography) revealed dilated bile ducts and a hypodense lesion in the left hepatic lobe, prompting a left hemihepatectomy. Histopathological analysis confirmed a 12 cm mucinous, papillary cholangiocarcinoma. Postoperative follow-up showed no recurrence or metastasis. Treatment strategies depend on disease extent, with surgical resection being the preferred approach for localized disease, while liver transplantation remains the only curative option for bilateral involvement.

## Introduction

Caroli's disease, first described by Jacques Caroli in 1958, is a rare congenital disorder that involves segmental cystic dilatations of the intrahepatic bile ducts [1]. These anomalies result from incomplete remodeling of the ductal plate during embryogenesis. The condition manifests as isolated Caroli's disease or Caroli's syndrome, with the latter including congenital hepatic fibrosis and portal hypertension. Caroli's disease predisposes patients to recurrent bacterial cholangitis, biliary lithiasis, and, in advanced stages, biliary cirrhosis. The syndrome, in particular, presents additional challenges due to the associated hepatic fibrosis [2, 3].

The risk of developing intrahepatic cholangiocarcinoma in patients with Caroli's disease is significantly elevated, with malignancy rates estimated between 7% and 16% [4]. This elevated risk underscores the importance of early detection and management. Imaging modalities such as magnetic resonance cholangiopancreatography (MRCP) and computed tomography (CT) play critical roles in identifying the characteristic cystic dilatations and associated complications [5, 6]. In recent years, advances in diagnostic imaging and surgical techniques have improved outcomes for patients diagnosed with this condition.

Although Caroli's disease is rare, it poses considerable diagnostic and therapeutic challenges due to its overlap with other biliary disorders, including primary sclerosing cholangitis and polycystic liver disease. Therapeutic approaches are largely dictated by the extent and localization of the disease. In cases with localized involvement, surgical resection, such as segmental liver resection, is the treatment of choice. For diffuse and bilateral disease, liver transplantation remains the only curative option [7, 8]. The condition is associated with high morbidity and mortality if not diagnosed and managed promptly.

This report discusses the case of a male patient in his 50s who presented with symptoms of Caroli's disease complicated by intrahepatic cholangiocarcinoma. It highlights the diagnostic challenges, therapeutic approaches, and outcomes to provide insights into the management of such complex cases.

## Case report

A male patient in his 50s presented to our specialized center with recurrent upper abdominal pain, chronic fatigue, and night sweats. These symptoms had progressively worsened over several months, prompting referral for further evaluation. His medical history included a cystic liver lesion diagnosed ~5 years earlier during imaging for unrelated reasons. No family history of liver diseases or malignancies was reported.

**Figure 1:**
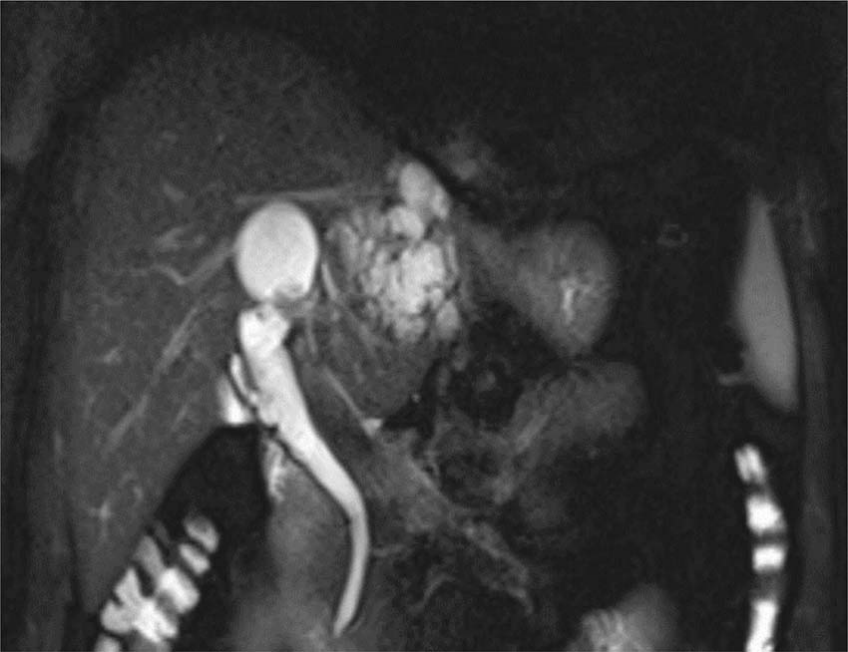
MRCP demonstrates multiple hyperintense, cystic lesions representing dilated intrahepatic bile ducts in the left hepatic lobe.

**Figure 2:**
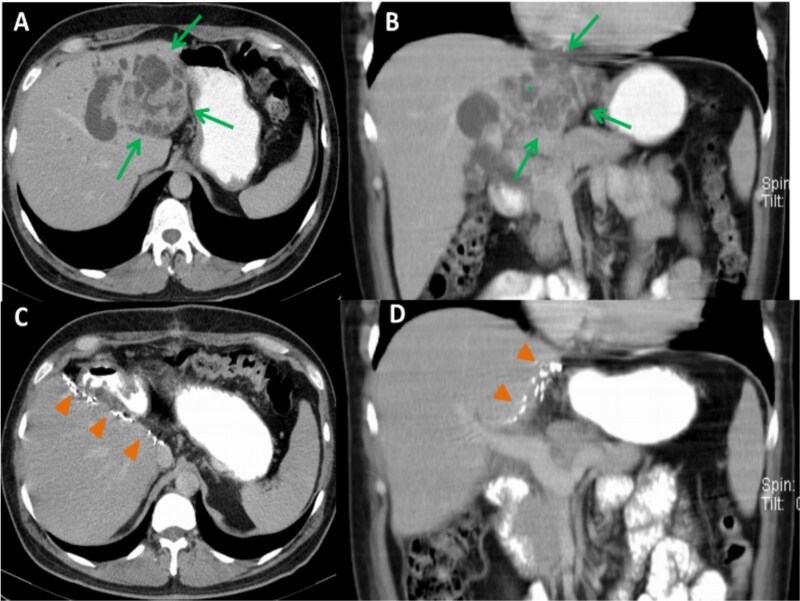
Preoperative CT images, axial (A) and coronal (B) planes, show a large, polycystic lesion in the left hepatic lobe (arrows), dilated intrahepatic bile ducts, and the “central dot” sign (*). Postoperative axial (C) and coronal (D) CT images after left hemihepatectomy show no focal lesion in the right hepatic lobe.

On clinical examination, mild tenderness in the upper abdomen was noted without jaundice or palpable masses. Laboratory investigations, including liver function tests, were within normal ranges, and no evidence of systemic infection was observed. Imaging studies using MRCP and CT demonstrated cystic dilatations of the intrahepatic bile ducts and a 5 cm hypodense lesion in the left hepatic lobe, raising suspicion for malignant transformation (Figs 1 and 2).

**Figure 3:**
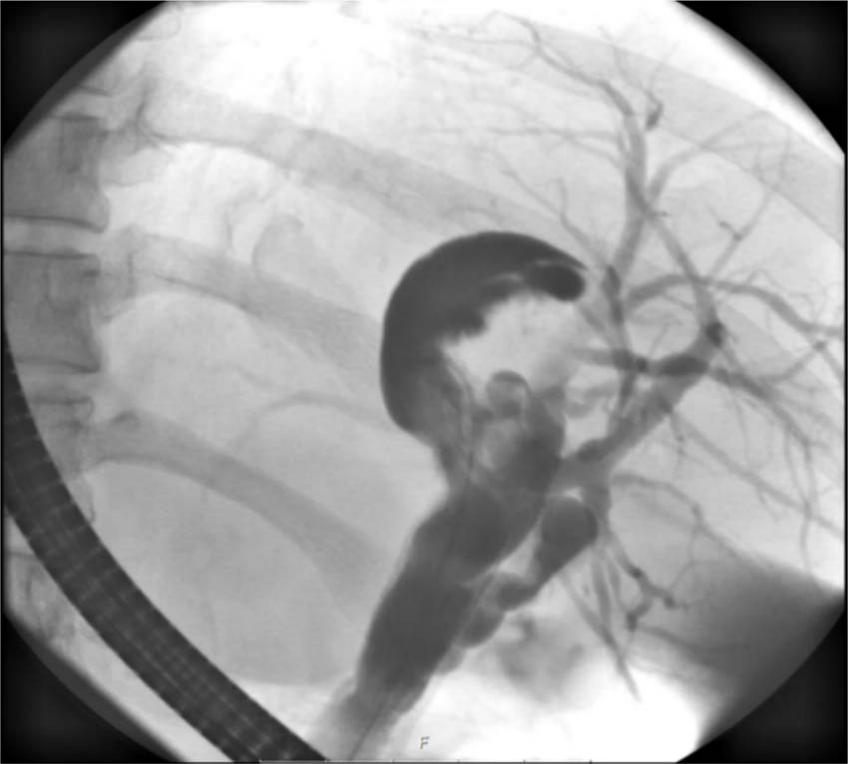
ERCP reveals a crescent-shaped, contrast-enhancing structure (40 mm in length) originating from the left hepatic duct.

Endoscopic retrograde cholangiopancreatography (ERCP) confirmed communication between the dilated bile ducts and the lesion in the left hepatic lobe (Fig. 3). There were no signs of biliary obstruction or extrahepatic involvement. Based on the suspicion of malignancy and the localized nature of the lesion, surgical intervention was planned.

**Figure 4:**
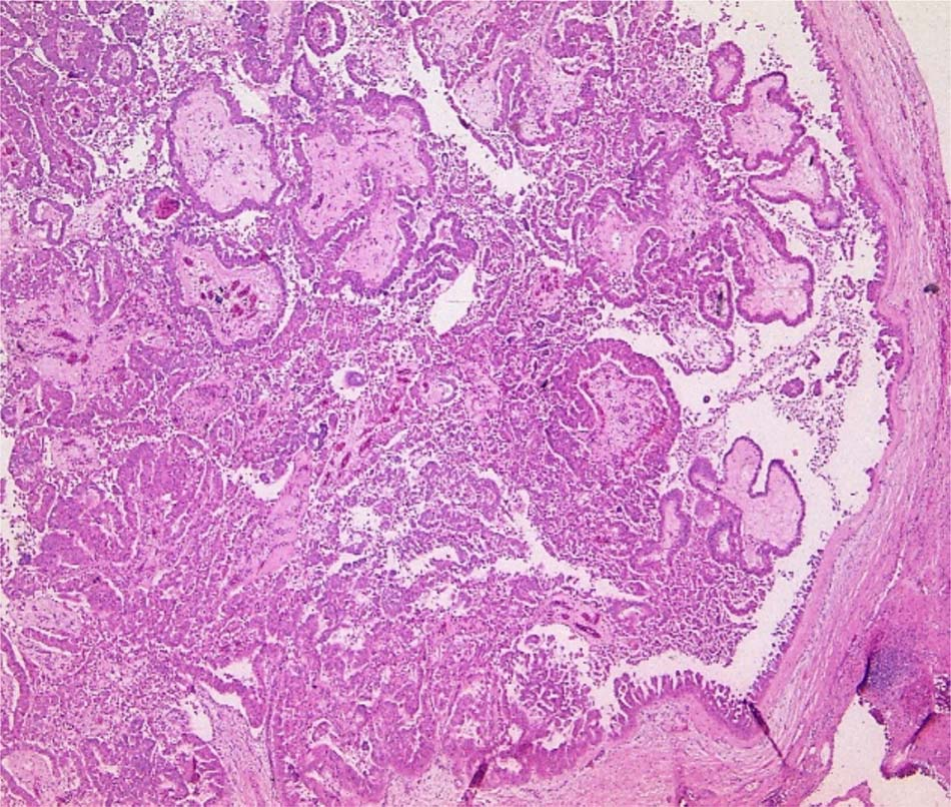
Pathology specimen shows markedly cystic dilated intrahepatic bile ducts with infiltrating adenocarcinoma.

A left hemihepatectomy was performed successfully. Intraoperative findings revealed a tumor confined to segments II and III of the liver. The resected specimen measured 12 cm in diameter. Histopathological analysis confirmed the diagnosis of intrahepatic cholangiocarcinoma associated with Caroli's disease (Fig. 4). The resection margins were free of tumor involvement.

The postoperative course was uneventful, and the patient was discharged after 2 weeks. Follow-up imaging at 3 and 6 months revealed no recurrence or metastasis. The patient reported significant symptom improvement and resumed normal activities.

## Discussion

Caroli's disease presents with a wide range of symptoms, from recurrent upper abdominal pain to severe complications such as biliary sepsis and liver abscesses. Differential diagnoses include primary sclerosing cholangitis, biliary atresia, and polycystic liver disease. These overlapping features emphasize the importance of comprehensive history-taking and targeted diagnostics.

Hepatic involvement may occur in isolation or in association with a genetic disorder, most commonly autosomal recessive polycystic kidney disease (ARPKD). However, there have also been reports of associations with autosomal dominant polycystic kidney disease. This raises uncertainty about whether all cases of Caroli’s disease are allelic to ARPKD [2, 9].

Imaging modalities such as MRCP and CT play a central role in identifying cystic dilatations and evaluating space-occupying lesions. MRCP provides non-invasive visualization of the bile duct system, while CT offers detailed information about tumor size and location [10]. ERCP serves additional diagnostic and therapeutic purposes, particularly in complicated cases of cholangitis [11]. Advanced imaging modalities, such as diffusion-weighted MRI, have shown promise in improving diagnostic accuracy and differentiation between benign and malignant lesions.

Therapeutically, surgical resection remains the standard treatment for localized malignant lesions. In this case, the left hemi-hepatectomy proved highly effective, as it enabled complete tumor removal with negative margins. Reports by Kassahun et al. and Mabrut et al. support this approach, showing that resection of localized lesions yields favorable long-term outcomes. Patients experiencing recurrent episodes of biliary infection, especially when accompanied by complications such as portal hypertension, may necessitate liver transplantation [12–14]. Genetic research is also beginning to illuminate pathways that may possibly provide targeted therapeutic approaches to address systemic involvement and improve long-term outcomes. Regular imaging follow-ups are essential to enable early intervention [15].

## Conclusion

This case enhances our understanding of Caroli's disease and its malignant transformations. It underscores the importance of a multidisciplinary approach, involving surgeons, radiologists, and oncologists, to optimize patient outcomes. Further research is warranted to evaluate long-term prognoses and innovative therapeutic strategies in managing this rare but complex condition.

## Data Availability

Data available on request. The data underlying this article will be shared on reasonable request to the corresponding author.
